# The Health Literacy Questionnaire (HLQ) at the patient-clinician interface: a qualitative study of what patients and clinicians mean by their HLQ scores

**DOI:** 10.1186/s12913-017-2254-8

**Published:** 2017-04-27

**Authors:** Melanie Hawkins, Stephen D Gill, Roy Batterham, Gerald R Elsworth, Richard H Osborne

**Affiliations:** 1 Faculty of Health, Centre for Population Health Research, Deakin University, Geelong, Australia; 2 Safety and Quality Unit, Barwon Health, Geelong, Australia; 3 Senior Research Fellow, Dhurakij Pundit University, Bangkok, Thailand

**Keywords:** Validity, Health Literacy Questionnaire, HLQ, Patient centred care, Patient reported outcomes

## Abstract

**Background:**

The Health Literacy Questionnaire (HLQ) has nine scales that each measure an aspect of the multidimensional construct of health literacy. All scales have good psychometric properties. However, it is the interpretations of data within contexts that must be proven valid, not just the psychometric properties of a measurement instrument. The purpose of this study was to establish the extent of concordance and discordance between individual patient and clinician interpretations of HLQ data in the context of complex case management.

**Methods:**

Sixteen patients with complex needs completed the HLQ and were interviewed to discuss the reasons for their answers. Also, the clinicians of each of these patients completed the HLQ about their patient, and were interviewed to discuss the reasons for their answers. Thematic analysis of HLQ scores and interview data determined the extent of concordance between patient and clinician HLQ responses, and the reasons for discordance.

**Results:**

Highest concordance (80%) between patient and clinician item-response pairs was seen in Scale 1 and highest discordance (56%) was seen in Scale 6. Four themes were identified to explain discordance: 1) Technical or literal meaning of specific words; 2) Patients’ changing or evolving circumstances; 3) Different expectations and criteria for assigning HLQ scores; and 4) Different perspectives about a patient’s reliance on healthcare providers.

**Conclusion:**

This study shows that the HLQ can act as an adjunct to clinical practice to help clinicians understand a patient’s health literacy challenges and strengths early in a clinical encounter. Importantly, clinicians can use the HLQ to detect differences between their own perspectives about a patient’s health literacy and the patient’s perspective, and to initiate discussion to explore this. Provision of training to better detect these differences may assist clinicians to provide improved care.

The outcomes of this study contribute to the growing body of international validation evidence about the use of the HLQ in different contexts. More specifically, this study has shown that the HLQ has measurement veracity at the patient and clinician level and may support clinicians to understand patients’ health literacy and enable a deeper engagement with healthcare services.

## Background

Data derived from patient-reported outcomes measures (PROMs) affect care decisions for individual patients through to decisions about nationwide health plans. Data are used to justify, endorse or exclude treatments, interventions and policies. Such responsibility requires the measurement tool and its data to be valid for the purpose [1, 2]. Meaning ascribed to data must be representative of the constructs the tool purports to measure, and the consequences of that interpretation must be valid for the intended purpose [2–7]. This means that validation of the data generated by a measurement tool is required for each new context in which it is used [2, 8].

As well as rigorous psychometric testing during the construction and initial validation of a questionnaire, it is also incumbent on researchers and decision-makers to demonstrate that the inferences made from questionnaire data are an acceptable representation of respondents’ real world situations within particular contexts. Whether measurement is to occur at the population level or at the individual level, or both, it is critical that the items measure what they intend to measure in all settings in which the questionnaire is applied. Construct validity relies on a questionnaire measuring what it purports to measure in all relevant contexts and, consequently, that the measurement of a particular construct can occur systematically among groups and settings [9–11].

Adamson and Gooberman-Hill [12] explored the meanings and interpretations behind people’s responses to commonly applied questions and questionnaires by eliciting narrative data from participants as they completed a questionnaire or set of questions. The narrative data revealed definitions and meanings of words and phrases in the questions that were different from the intention of the items. The study demonstrated that items are often not clear, precise and brief, and that double or ambiguous meanings can be embedded within an item. Validity relies on respondents’ collective understanding of items and the associated response options, and consequently that respondents with similar characteristics, in relation to the construct being measured, will systematically respond to items in the same way [3]. This also suggests that there will be idiosyncratic variations in the interpretation of individual questions by individual people and that, even when aggregate data can be safely interpreted for a population, considerations must be applied when interpreting and making decisions based on scores from individuals. Many PROMs, including recent health literacy PROMs, have been designed and tested for use at the population level, and have not been tested for use with individual patients [13, 14].

Measurement of health literacy has proved complex because it is a multidimensional concept and definitions for it have evolved over many years [15, 16]. The World Health Organization definition of health literacy was used for the development of the Health Literacy Questionnaire (HLQ) [14]: health literacy ‘…is the cognitive and social skills which determine the motivation and ability of individuals to gain access to, understand and use information in ways which promote and maintain good health’ [17]. While the purpose of this definition is to convey the broad meaning of the concept to researchers, practitioners, policymakers and others, it is not a concept that is easy to capture and measure at the individual person level. Consequently, development of the HLQ used a validity-driven approach [1, 14] with extensive patient engagement, including during the conceptual development of constructs and items, and for the cognitive testing of items.

The HLQ was designed using a grounded, validity-driven approach and initially tested in diverse samples of individuals in Australian communities and is shown to have strong construct validity, reliability and acceptability to clients and clinicians [14, 18, 19]. The HLQ measures nine independent domains of health literacy to capture the lived experiences of people attempting to understand, access and use health information and health services. The scales generate profiles of individuals, groups and populations. Importantly, the data also reflect the quality of health and social service provision. Service providers can use the profiles to better understand the needs of communities, and assist with planning, designing and evaluating interventions. It was designed for self-administration using pen and paper and can also be interviewer-administered to ensure inclusion of people who cannot read or have other difficulties with self-administration.

The HLQ is used in many countries and in many settings, including for population health surveys [20], development of interventions [19], and for evaluation of health programs [21, 22]. Validation of the interpretation of data for an intended purpose is recommended for each new setting [1, 2]. Osborne et al support a validity-driven approach to the validation of the data derived from measurement tools, stating that the HLQ is ‘now ready for further testing and validation of the interpretations of each scale’s data in the intended application settings; that is, applications in specific demographic groups, within health promotion, public health and clinical interventions, and in population health surveys’ (p.13) [14]. If individual patient HLQ data are to be interpreted and used by clinicians to make decisions about treatment for those patients then validation of patient and clinician interpretations of HLQ data must be undertaken.

The purpose of this study was to establish the extent of concordance and discordance between patient and case manager (clinician) HLQ scores and the corresponding interview narratives (interpretations of those scores) across the nine independent HLQ scales, and to identify the reasons for discordance. To do this, the study examined interpretations of HLQ item scores in a setting with individual patients who had chronic and complex health conditions, who were participating in intensive case management, and whose clinician thought were likely to have low health literacy. Both the patient and their clinician completed the HLQ and were interviewed, and the data compared. If some systematic discordance exists between patient and clinician interpretations of HLQ scores, and this is known, then clinicians will be able to use the HLQ data in a more informed way in support of clinical practice.

The study sought to answer the following research questions:

1. What do patients really mean by their HLQ scores? That is, how well do patients’ HLQ scores match their interview narrative data?
2. What is the extent of concordance between patients’ HLQ scores and narratives and their clinician’s HLQ scores and narratives about the patients, and what are the reasons for discordance?

The first of these questions directly addresses validation of HLQ data for individual patients (not populations) within a chronic and complex care context, and contributes to the ongoing development of the web of evidence about the HLQ and its clinical and public health utility. The second question addresses the concordance of patients’ perspectives with their clinicians’ perspectives to determine the utility of the HLQ as a tool to inform clinicians about their patients’ health literacy needs, and to facilitate discussions with patients when HLQ scores differ from clinicians’ expectations.

## Methods

### Study design

A qualitative design using HLQ scores and semi-structured interviews was employed so that interview narratives revealed patient and clinician experiences and reasons behind why they chose particular HLQ scores. Patient and clinician data were assessed for match between HLQ scores and corresponding interview narratives, and then for concordance and discordance between patient and clinician score/narrative responses. Patient and clinician data were analysed thematically across HLQ scales to determine the extent of concordance between patient and clinician HLQ responses (scores and narratives), and the reasons for discordance.

### Setting

The study was conducted at a large regional Australian public health service, Barwon Health, which comprises a range of community care services and a major teaching hospital. Staff and patients were recruited from the organisation’s Hospital Admission Risk Program (HARP), an intensive case management service to support people who have complex and chronic conditions and/or frequently attend emergency departments. In this service, clinicians come to know their patients very well, including their personal and domestic situations, through home visits and attending medical appointments with them.

### Participants

A priority for this study was to include individuals who might have low health literacy, which is a group often overlooked, omitted or missed in research projects, usually because they are difficult to engage. This is often the case for clients assigned to the HARP service and was the reason this site was chosen for recruitment. People with higher health literacy are more likely to be well educated and competent in accessing health care and in answering questionnaires, and are likely to more strongly endorse the items of the HLQ (i.e., answer Strongly Agree and Very Easy). In order for this study to be more likely to rigorously explore the depth and breadth of the HLQ constructs – and therefore to test the validity of the HLQ data in this individual patient context – all existing patients of the participating HARP clinicians who met the criteria were recruited to the study. A high response rate from this group of patients was not expected so, as HLQs were returned, all who met the criteria were included.

HARP clinicians were specifically requested, based on their extensive knowledge of their clients, to deliberately include clients who they thought may have health literacy difficulties. Inclusion criteria for participants included engagement for four or more months in HARP case management and care coordination, a comprehensive HARP assessment, and at least six contacts with the HARP clinician. This criteria maximises the opportunity for the clinician to get to know the patient well and, as such, respond to HLQ items about a patient in a way that is reflective of that patient’s health context. It was a way of confirming patients’ HLQ responses in the absence of external data about the patients’ actual lived experiences. Patients were invited to participate in the study by their HARP clinician. The professions of the clinicians included nursing, social work and dietetics.

### Ethics

The project was approved by the Human Research Ethics Committees of Barwon Health (ID: 11/85) and Deakin University (ID: 2011-077).

### Data collection

Consenting patients either self-completed the HLQ or were assisted by a friend, relative or carer (but not their HARP clinician). Demographic and health data were also collected from the patients. Clinicians were asked to complete the HLQ about their patient in two ways: first, from their own perceptions of the patient’s health literacy status and, second, how they think their patient would respond to the items. This paper reports only on the comparison of patient scores with the first set of scores from each clinician’s perspective of their patient’s health literacy.

Semi-structured telephone interviews were conducted by authors MH or SG. Most interviews were conducted between 3 and 8 weeks after an HLQ was completed by the patient. Interviews consisted of reading HLQ questions to patients and clinicians, reminding them of the answer they had given to that item, then prompting with questions such as ‘Can you tell me why you chose that answer?’ and ‘What were you thinking about when you selected that answer?’. The interviewers did not inform clinicians of their patient’s scores during the clinician interviews.

Development and validation of the HLQ is described elsewhere [14]. The development and validation study showed the HLQ has strong construct validity, reliability and acceptability to clients and clinicians [14, 18, 19]. The original scale reliability estimates ranged from 0.77 to 0.90 [14], and were reproduced in a more diverse replication sample with scores ranging from 0.80 to 0.89 [23]. The a priori 9-factor structure was confirmed in both the original development study and the replication study. Detailed analysis of the relationships between the health literacy scales and socioeconomic position in the vulnerable groups demonstrated expected small to large associations with key demographic factors [24]. Table 1 displays the high and low descriptors and psychometric properties for each of the nine HLQ scales [14]. Each of the nine scales comprise between 4 and 6 items (44 items in total). Each item has a corresponding description of the meaning and intent of the item, which supports the purpose and positioning of the item within the scale. Items are scored from 1-4 in the first 5 scales (Strongly Disagree, Disagree, Agree, Strongly Agree), and from 1-5 in scales 6-9 (Cannot Do, Very Difficult, Quite Difficult, Easy, Very Easy). HLQ validation and testing included extensive cognitive testing to confirm the items were understood as intended.

**Table 1:** High and low descriptors and psychometric properties of HLQ scales

Low level of the construct	High level of the construct
Scale 1. Feeling understood and supported by healthcare providers	
People who are low on this domain are unable to engage with doctors and other healthcare providers. They don’t have a regular healthcare provider and/or have difficulty trusting healthcare providers as a source of information and/or advice.	Has an established relationship with at least one healthcare provider who knows them well and who they trust to provide useful advice and information and to assist them to understand information and make decisions about their health.
Psychometric properties: Model Fit – *χ* ^2^ WLSMV(2) = 10.15, *p* = 0.0063, CFI = 0.998, TLI = 0.995, RMSEA = 0.100, and WRMR = 0.367. Composite reliability = 0.88 (0.86-0.90)	
Scale 2. Having sufficient information to manage my health	
Feels that there are many gaps in their knowledge and that they don't have the information they need to live with and manage their health concerns.	Feels confident that they have all the information that they need to live with and manage their condition and to make decisions.
Psychometric properties: Model Fit – *χ* ^2^ WLSMV(2) = 5.24, *p* = 0.0730, CFI = 1.000, TLI = 0.999, RMSEA = 0.063, and WRMR = 0.337. Composite reliability = 0.88 (0.87-0.90)	
Scale 3. Actively managing my health	
People with low levels don’t see their health as their responsibility, they are not engaged in their healthcare and regard healthcare as something that is done to them.	Recognise the importance and are able to take responsibility for their own health. They proactively engage in their own care and make their own decisions about their health. They make health a priority.
Psychometric properties: Model Fit – *χ*2 WLSMV(5) = 31.96, p < 0.0001, CFI = 0.992, TLI = 0.983, RMSEA = 0.115, and WRMR = 0.775. Composite reliability = 0.86 (0.84-0.88)	
4. Social support for health	
Completely alone and unsupported for health.	A person’s social system provides them with all the support they want or need for health.
Psychometric properties: Model Fit – *χ* ^2^ WLSMV(5) = 37.36, *p* < 0.0001, CFI = 0.987, TLI = 0.975, RMSEA = 0.126, and WRMR = 0.925. Composite reliability = 0.84 (0.81-0.86)	
5. Appraisal of health information	
No matter how hard they try, they cannot understand most health information and get confused when there is conflicting information.	Able to identify good information and reliable sources of information. They can resolve conflicting information by themselves or with help from others.
Psychometric properties: Model Fit – *χ* ^2^ WLSMV(5) = 18.05, *p* = 0.0029, CFI = 0.990, TLI = 0.980, RMSEA = 0.080, and WRMR = 0.610 Composite reliability = 0.77 (0.74-0.81)	
6. Ability to actively engage with healthcare providers	
Are passive in their approach to healthcare, inactive i.e., they do not proactively seek or clarify information and advice and/or service options. They accept information without question. Unable to ask questions to get information or to clarify what they do not understand. They accept what is offered without seeking to ensure that it meets their needs. Feel unable to share concerns. The do not have a sense of agency in interactions with providers.	Is proactive about their health and feels in control in relationships with healthcare providers. Is able to seek advice from additional healthcare providers when necessary. They keep going until they get what they want. Empowered.
Psychometric properties: Model Fit – *χ* ^2^ WLSMV(5) = 74.91, *p* < 0.0001, CFI = 0.986, TLI = 0.973, RMSEA = 0.185, and WRMR = 0.944. Composite reliability = 0.90 (0.88-0.92)	
7. Navigating the healthcare system	
Unable to advocate on their own behalf and unable to find someone who can help them use the healthcare system to address their health needs. Do not look beyond obvious resources and have a limited understanding of what is available and what they are entitled to.	Able to find out about services and supports so they get all their needs met. Able to advocate on their own behalf at the system and service level.
Psychometric properties: Model Fit – *χ* ^2^ WLSMV(9) = 21.74, *p* = 0.0097, CFI = 0.998, TLI = 0.996, RMSEA = 0.058, and WRMR = 0.451. Composite reliability = 0.88 (0.87-0.90)	
8. Ability to find good health information	
Cannot access health information when required. Is dependent on others to offer information.	Is an 'information explorer'. Actively uses a diverse range of sources to find information and is up to date.
Psychometric properties: Model Fit – *χ* ^2^ WLSMV(5) = 57.06, *p* < 0.0001, CFI = 0.989, TLI = 0.977, RMSEA = 0.160, and WRMR = 0.820. Composite reliability = 0.89 (0.87-0.91)	
9. Understand health information well enough to know what to do	
Has problems understanding any written health information or instructions about treatments or medications. Unable to read or write well enough to complete medical forms.	Is able to understand all written information (including numerical information) in relation to their health and able to write appropriately on forms where required.
Psychometric properties: Model Fit – *χ* ^2^ WLSMV(5) = 35.70, *p* < 0.0001, CFI = 0.992, TLI = 0.983, RMSEA = 0.123, and WRMR = 0.671 Composite reliability = 0.88 (0.86-0.90)	

Adapted from Osborne et al, [14]

### Data analysis

In this study, a ‘patient-clinician dyad’ refers to a patient and that patient’s clinician. To reduce interviews to a manageable length, dyads were administered subsets of the nine scales. Dyads were alternately assigned to each group as completed HLQs were received. Group 1 consisted of Scales 1, 2, 3 (Disagree/ Agree response options), 6 and 7 (Difficult/ Easy response options). Group 2 consisted of Scales 4, 5 (Disagree/ Agree response options), 8 and 9 (Difficult/ Easy response options). Data were collected from 9 dyads for Group 1 and 7 dyads for Group 2. A ‘patient-clinician item-response pair’ refers to a patient’s HLQ score and interview narrative paired with the corresponding clinician’s HLQ score and interview narrative *for one HLQ item*. For reporting purposes, patients and clinicians are identified with a P or C, respectively, and their study number (for example, P101 and C101).

Data analysis was two-fold: 1) determine if interview narrative data were consistent with patients’ and clinicians’ HLQ scores (and if the narrative reflected the intent of the items); and 2) determine the extent of concordance (and discordance) between patient HLQ scores and narratives and clinician HLQ scores and narratives (that is, the extent of concordance within patient-clinician item-response pairs).

In the first step, patient and clinician data were examined separately. To assist researchers’ understanding of items and scales, and to guide the linguistic and cultural adaption of items to other languages and cultures, a short description of each item has been written to explain what the item intends to convey (and not to convey). These item intents are part of the HLQ support documentation. In the current study, the first step for both patient and clinician data was to compare an HLQ score (e.g., Agree or Always Easy to do) with the corresponding narrative to assess if the narrative made sense in light of the score (i.e., if it matched the score) and the item intent. For example, if a score was that a task was ‘Always Easy’ then the narrative was examined for confirmation that the respondent agreed with this score and/or a description of how or why it was always easy to do. A score and narrative were considered a match if the narrative indicated that the respondent agreed with the score they had assigned to an item, and the interview narrative matched the intent of the item. Accordingly, a score and narrative did not match if the narrative did not provide a statement that clearly demonstrated support for the score. Although this analysis was conducted on both patient and clinician data, only patient data from this step was required to answer the first research question. Clinician data was examined only to confirm match for the purposes of answering the second research question.

For the second step, patient HLQ scores *and* interview narratives were compared with their clinician’s HLQ scores *and* interview narratives (for each item) to determine the extent of concordance within patient-clinician item-response pairs across items within each HLQ scale. There were three ways that these data were categorised: 1) concordant, 2) discordant, or 3) unclear (that is, concordance or discordance could not be assigned to a patient-clinician pair because the patient or the clinician narrative did not match their corresponding score, or the patient or clinician changed their score during interview). Descriptions of the requirements for these categories are in Table 2.

**Table 2:** Requirements for concordance, discordance and unclear categories

	Requirement 1	Requirement 2	Requirement 3
Concordance	Patient’s narrative supports the HLQ score	Clinician’s narrative supports the HLQ score	Patient and clinician HLQ scores are on the same side of the response options scale
Discordance	Patient’s narrative supports the HLQ score	Clinician’s narrative supports the HLQ score	HLQ scores are on opposite sides of the response options scale
Unclear	Patient’s narrative does not support HLQ score or…	…clinician’s narrative does not support HLQ score or…	…patient or clinician changed the score during interview.

Each HLQ scale comprised between 4 and 6 items with data collected for 7 or 9 dyads per scale (i.e., from 35 to 63 patient-clinician item-response pairs across the 9 scales), such that there was a total of 408 item-response pair interactions. Two researchers (MH and SG) independently examined all HLQ scores and corresponding narrative data and then sought consensus, including specific reasons for concordance, discordance, and unclear responses. Data were then reanalysed to confirm boundaries and categories for concordance, discordance, and unclear pairs. Analysis of interview narratives included initial coding of narratives for match with corresponding HLQ scores and for reasons why a score was chosen; categorisation of narratives to determine common reasons for choice of scores within scales; and then thematic analysis of these categories across patient-clinician item-response pairs for common themes for discordance across scales [25, 26].

Patient and clinician HLQ scores located on the same side of the response option scale (e.g., Cannot Do and Quite Difficult, or Agree and Strongly Agree) were classified as concordant, whereas score pairs located at opposing ends of the response option scale (e.g., Disagree and Agree) were classified as discordant.

Forty-five HLQs were distributed to HARP patients, of which 22 were returned, and full consent was received by 20 of those. Interviews were conducted with 18 patients because 2 were subsequently unable to be contacted. There were 2 patients who were particularly difficult to contact and were interviewed 12 weeks (P114) and 21 weeks (P104) after returning their HLQs. HARP clinicians needed to facilitate the contact between these patients and the researchers, with one patient preferring to be interviewed face-to-face. There were 9 clinicians interviewed, each of whom were responsible for between 1 and 4 patients. Overall, both HLQ scores and narrative data were collected for 16 patient-clinician dyads.

## Results

Demographic characteristics for patients are shown in Table 3. The median age of the 16 patients was 43 years (range 18-77; SD 18) with 11 people under 55 years. There were 10 females, 7 participants did not complete high school, 13 lived alone, 15 spoke English at home, 13 were born in Australia, and 6 had four or more chronic conditions.

**Table 3:** Demographic data for patients interviewed (*N* = 16, except for age and lung disease *N* = 15)

	*N (%)*
Female	10 (63%)
Age ≥55 years	4 (27%)
Lives alone	13 (81%)
Did not complete high school	7 (44%)
Born in Australia	13 (81%)
English spoken at home	15 (94%)
Identifies as Indigenous/Torres Strait Islander	0 (0%)
Arthritis/musculoskeletal condition	5 (31%)
Back Pain	7 (44%)
Heart disease	6 (38%)
Lung disease	5 (33%)
Cancer	1 (6%)
Depression/Anxiety	9 (56%)
Diabetes Mellitus	7 (44%)
Stroke/neurological condition	3 (19%)
≥4 chronic conditions	6 (38%)
Private Health Insurance	3 (19%)
Received government benefits (aged pension or disability)	16 (100%)
Assistance with questionnaire	1 (6%)

The majority of the 38 unclear patient-clinician item-response pairs were because clinicians changed their scores during the interview (13 changes across 6 clinicians) with this followed closely by patient narratives that did not support the HLQ scores (12 non-matches across 6 patients). There were 9 instances (also across 6 patients) when patients changed their scores during the interview (only 1 of these patients had also provided a narrative that did not match the score). There were 4 instances (across 3 clinicians) when a clinician’s narrative did not support the HLQ (2 of these clinicians also changed a score during interview).

Given that some clinicians completed HLQs and were interviewed about more than one patient, it was possible that the data may have revealed clinician response patterns. However, systematic assessment of the data from each clinician could find no evidence of response patterns for any one clinician.

1. *What do patients really mean by their HLQ scores? That is, how well do patients’ HLQ scores match their narrative data?* (Patient data only)

Overall and across scales, patient interview narratives gave clear reasons to support the chosen response options, and these reasons reflected the intention of the HLQ items. Table 4 shows the match between patient scores and narratives for items across the nine HLQ scales.

**Table 4:** Match (step 1: patient score + narrative); concordance, discordance and unclear (step 2: patient and clinician score + narrative)

HLQ scale	Patient-clinician dyads per scale (N)	Items per scale	Total patient-clinician item-response pairs	N (%) Match (step 1)	N (%) Concordance (step 2)	N (%) Discordance (step 2)	N (%) Unclear (step 2)
**1.** Feeling understood and supported by healthcare providers	9	6	54	51 (94%)	43 (80%)	8 (15%)	3 (5%)
**2.** Having sufficient information to manage my health	9	7	63	62 (98%)	38 (60%)	24 (38%)	1 (2%)
**3. **Actively managing my health	9	5	45	41 (91%)	31 (69%)	10 (22%)	4 (9%)
**4.** Social support for health	7	5	35	35 (100%)	17 (49%)	17 (49%)	1 (3%)
**5.** Appraisal of health information	7	6	42	41 (98%)	28 (67%)	10 (24%)	4 (10%)
**6.** Ability to actively engage with healthcare providers	9	5	45	45 (100%)	19 (42%)	25 (56%)	1 (2%)
**7.** Navigating the healthcare system	9	6	54	48 (89%)	32 (59%)	14 (26%)	8 (15%)
**8.** Ability to find good health information	7	5	35	30 (86%)	15 (43%)	12 (34%)	8 (23%)
**9.** Understand health information well enough to know what to do	7	5	35	34 (97%)	14 (40%)	13 (37%)	8 (23%)

Two patients exhibited some difficulty with some items. P114 had several co-morbidities, exhibited confusion during the interview, and had difficulty concentrating on items and providing answers. P115 changed her responses for 4 of the 5 items in scale ‘8. Ability to find good health information’ from the ‘Difficult’ end of the response options scale to the ‘Easy’ end. She seemed unsure as to why she had originally answered that these tasks were difficult. These two participants contributed to scale ‘8. Ability to find good health information’ having the lowest match between patient scores and narratives (30 of the 35 responses [7 patients x 5 items] for that scale), but still high at 86%.

For scale ‘4. Social support for health’ and ‘6. Ability to actively engage with healthcare providers’, all patient narratives clearly supported the corresponding HLQ scores. There were no unclear narratives, no opposing narratives, and no patients changed their answers during the interviews.

2. *To what extent are patients’ HLQ scores concordant with those provided by their clinician, and what are the reasons for discordance?* (Patient and clinician data)

The number of concordant, discordant and unclear patient-clinician item-response pairs across HLQ scales is shown in Table 4.

Highest concordance between patient and clinician item-response pairs was seen in ‘1. Feeling understood and supported by healthcare providers’ (80%). Highest discordance (56%) was seen in ‘6. Ability to actively engage with healthcare providers’. Lowest concordance (given the unclear category) was 40% for ‘9. Understand health information well enough to know what to do’, closely followed by ‘6. Ability to actively engage with healthcare providers’ (42%) and ‘8. Ability to find good health information’ (43%). Three scales had 8 unclear patient-clinician item-response pairs: ‘7. Navigating the healthcare system’, ‘8. Ability to find good information’ and ‘9. Understand health information well enough to know what to do’.

### Concordance

Concordance means that both patients and clinicians perceived that the patient had or did not have resources or skills (e.g., was able to form relationships), or could or could not do certain tasks (e.g., fill in medical forms). That is, both respondents scored (with narratives supporting this score) on the same side of the response options scale. In the following example, both patient and clinician scored Agree in response to an item about her relationships with healthcare providers, and their narratives support their scores. P108 (HLQ response option selected = Agree) said *my GP for instance has phoned me at home and followed up on a couple of things and actually saved my life once by doing so, so I trust her*. Her clinician C108 (HLQ response option selected = Agree) said *I’ve been to the GP with this client and she has a long relationship with the GP and a fond relationship with the GP*. See Table 5 for more examples of concordance.

**Table 5:** Examples of patient-clinician concordance

HLQ scales	Patients	Clinicians
Scale 1. Feeling understood and supported by healthcare providers	P103 (Agree) *I've got diabetes so I go to the diabetes referral centre at the hospital and my GP and all that. And the woman from HARP so there's, like, a lot of supportive people.*	C103 (Agree) *He knows where to go to get the support he needs.*
Scale 2. Having sufficient information to manage my health	P108 (Strongly Agree) *Yes, I strongly agree because of my background [nursing]…and I'm not afraid to ask providers 'what's this?' and 'how does that work?' and 'why isn't that done?' and what have you. That's the reason I strongly agree with that. I can do that.*	C108 (Strongly Agree) *Yes…because of her professional background. She has a good understanding of the medical system and seeks information from various sources.*
Scale 3. Actively managing my health	P105 (Disagree) *I don’t do everything that I should… I still smoke and still have a couple of beers. That doesn't help.*	C105 (Disagree) *…from what I've witnessed he drinks beer and smokes cigarettes and sits on his couch for 8 h a day or on the Internet and literally that's all I've seen him do.*
Scale 4. Social support for health	P113 (Agree) *I can have either my father…or [HARP clinician] will come… It's pretty easy…The only reason I wouldn't have gone 'strongly agree' is sometimes they're busy or other people are busy and they can't always be there when I'm really sick quite instantly with something.*	C113 (Agree) *Yes. Me and his father.*
Scale 5. Appraisal of health information	P115 (Strongly Disagree) *I don't look at health information so I can't really compare if I don't have it.*	C115 (Disagree) *She’ll be given a piece of information by one of the other residents and she won’t necessarily seek out another option or opinion from someone else to compare with what she has been told by a resident.*
Scale 6. Ability to actively engage with healthcare providers	P114 (Quite Difficult) *It’s always difficult because like I said they have quite a few dozen other people that they care for and they only have a short time to assess or discuss things with me.*	C114 (Quite Difficult) *There are times when he comes back and says 'you know, I wasn’t able to talk about that'. Sometimes the HCP also cuts him short because, you know, they've got a time limit. So it's being able to discuss all those issues that he might have had. We've tried to do lists and things for him to take a list along of the issues to try to keep him on track. But yeah, that's a struggle for him.*
Scale 7. Navigating the healthcare system	P122 (Very Easy) *The information I get from Barwon Health and the GP, it seems to be all provided for me.*	C122 (Quite Easy) *Because he gets guidance it is quite easy. Guidance from his health professionals, family and peers.*
Scale 8. Ability to find good health information	P111 (Cannot Do) *Because I don’t leave my unit and I don’t have access to a computer.*	C111 (Quite Difficult) *Yeah, and maybe that should really be VD by herself, and that means her accessing it herself, that would be very difficult I think.*
Scale 9. Understand health information well enough to know what to do	P104 (Cannot Do) *I give my best. Sometimes it's difficult…You get these big words and think 'what are they talking about?'*	C104 (Very Difficult) *He would not be a candidate for any more information than maybe grade 5…very basic. And that goes for oral information and written information. It has to be broken down into very basic little chunks.*

### Discordance

Four main themes were identified for discordance between patient and clinician data across HLQ items.

1. Technical or literal meaning of specific words
2. Patients’ changing or evolving circumstances
3. Different expectations and criteria for assigning HLQ scores
4. Different perspectives about a patient’s reliance on healthcare providers

Some examples of these themes are presented in the results. See Table 6 for further examples.

**Table 6:** Examples of patient-clinician discordance for Themes 3 and 4

Theme 3. Different expectations and criteria for assigning HLQ scores	Patients	Clinicians
*Sub-theme 3a) Action is a more important criteria for clinicians than for patients*		
Scale 3. Actively managing my health	P105 (Agree) *There is not much else to do except to try to get better.*	C105 (Strongly Disagree) *He actually doesn’t do anything that he says he might do.*
Scale 9. Understand health information well enough to know what to do	P115 (Quite Easy) *They tell you what to do.*	C115 (Quite Difficult) *I don’t know whether it is more that she is just not willing to follow the advice. She may understand it but is not willing to act on it.*
*Sub-theme 3b) Patients don’t always know what they don’t know*		
Scale 6. Ability to actively engage with healthcare providers	P103 (Quite Easy) *If I don’t tell them [doctors] my problems, how can I expect them to give the right advice or help me? If I’m not honest, what’s the point in going?*	C103 (Quite Difficult) *I think it almost might be Very Difficult, he has limited understanding about his difficulties and finds it difficult to help doctors to understand. He is not insightful about his health issues.*
Scale 6. Ability to actively engage with healthcare providers	P112 (Quite Easy) *Yep. Most doctors are quite understanding.*	C112 (Quite Difficult) *Because she doesn’t ask prying questions. She will be told the information but doesn’t have a discussion. She might check it on the Internet but doesn’t talk with doctors.*
Scale 6. Ability to actively engage with healthcare providers	P116 (Very Easy) *I can pretty much discuss anything.*	*C116 (Quite Difficult) She couldn’t sit with the discomfort about what she needed to do. She wanted to ignore her condition and pretend it would go away. She wanted to distract or make a joke if the talk got too serious.*
Scale 9. Understand health information well enough to know what to do	P113 (Quite Easy) *That’s easy, no problem*	C113 (Quite Difficult) *I’m not sure what his comprehension is but I think it is not that high. He gets mixed up a bit with fairly simple instructions about how many times to take a tablet.*
*Sub-theme 3c) There are different points of comparison (providers compare across patients, patients compare across providers)*		
Scale 7. Navigating the healthcare system	P116 (Quite Easy) [About knowing the best care for her] *It’s the same thing every time; the right medication, 10 days in hospital on this drug, and then home.*	C116 (Quite Difficult) *Back then her decision making wasn’t that great. She would head for the hospital. It was all very reactive – wait till she gets sick then get help.*
*Sub-theme 3d) There are different expectations for support when ill*		
Scale 4. Social support for health	P102 (Strongly Disagree) *People say they’ll help but when the time comes, they don’t.*	C102 (Agree) *[She has] a lot of health practitioners and she’s got her mother, she can rely on her mother.*
Scale 4. Social support for health	P107 (Agree) *I have support from everybody… they come running.*	C107 (Disagree) *If they have a fight then they drop right off. Support is a bit* ad hoc *and always a drama. It is not strong support because it is not consistent.*
Scale 4. Social support for health	P111 (Strongly Agree) *Well, I ring an ambulance if I need help.*	C111 (Disagree) *She has some but she would really have her mother as her main support.*
Theme 4. Different perspectives about a patient’s reliance on healthcare providers	Patients	Clinicians
Scale 7. Navigating the healthcare system	P116 (Quite Easy) *But only though my carer lady [HARP clinician]*	C116 (Quite Difficult) *HARP can tell her. She wouldn’t go to the library, wouldn’t look it up on the computer.*
Scale 8. Ability to find good health information	P104 (Quite Easy) *I get it straight from the doctor’s surgery. He’s got boards up [with information].*	C104 (Quite Difficult) *The response would be Cannot Do if he was on his own but with the help of the healthcare providers he can do it.*

### Theme 1. Technical or literal meaning of specific words

In some cases, discordance related to specific words such as ‘sure’, ‘all’ and ‘plenty’. Patients did not comment on these words specifically. Clinicians, however, when thinking about a patient, sometimes read these words in a literal sense. While patient P103 (Agree) said he had all the information he needed (‘2. Having sufficient information to manage my health’), clinician C103 rated the item as Disagree explaining: *I guess it was in regards to the wording of ‘being sure’; it’s an absolute sort of word and so I think that is why I’ve done that again because of being 100% sure about something. I’m not sure that he might have all the information he needs*. A second example again shows how the clinicians notice the qualifier words and adjust their response accordingly. P113 disagreed with an item that asked about having plenty of people to rely on (‘4. Social support for health’), but his clinician (C113, Agree) stated *I wouldn’t say ‘plenty’ but the ones he’s got would be very reliable if he needs help*.

### Theme 2. Patients’ changing or evolving circumstances

Theme 2 is about patients who are learning to trust new healthcare providers and learning, over a period of time, to understand their own health conditions. This theme was categorised separately from themes 3 and 4 because of the specific context of patients’ relationships and understanding about their health being in a state of flux. Themes 3 and 4 are related to more stable and ongoing health contexts, and established and ongoing relationships with and reliance on healthcare providers.

In ‘1. Feeling understood or supported by healthcare providers’, patient-clinician perspectives differed around trusting healthcare providers when relationships with healthcare providers were new, evolving or changing. P112 described how she was recently establishing new relationships with healthcare providers, was learning to trust them and discuss her health with them: *I’ve only over the last year got certain, I suppose you could say ‘go-to people’ for my healthcare needs…I don't have anybody to discuss specific issues with…I'm finding people that I can trust with my health issues as well, because I've had a lot issues with that in the past, finding people that I can trust to deal with my health issues* (P112, Disagree). C112 scored Agree and, referring to these recently forming relationships with healthcare providers, explained: *Yes, she does have a healthcare person that she can speak with; whether she does or not is another matter.*

Some patients reported that their knowledge and understanding about their health was evolving (often because of previous lack of access to health information and care) and that they did not yet know all they would eventually know. In ‘2. Having sufficient information to manage my health’, P101 (Disagree) stated: *I don’t think I’ve got enough information at all*. C101 (Agree) said the patient had the information but because of ambivalence and some medication issues she didn’t deal with it well.

### Theme 3. Different expectations and criteria for assigning HLQ scores

This theme encompasses four overlapping sub-themes that reflect differences between patients and clinicians when it comes to assigning scores to the way patients respond to the provision of health information and services or health support: a) Action is a more important criterion for clinicians than for patients; b) Patients don’t always know what they don’t know; c) There are different points of comparison (providers compare across patients, patients compare across providers); and d) There are different expectations for support when ill.

### Sub-theme 3a) Action is a more important criteria for clinicians than for patients

Clinicians tended to expect to see patients take action to improve their health and often applied this criteria when answering the HLQ items. For example, although patients may have information about their health, clinicians sometimes determined that they didn’t always have the capacity to understand, retain or, in particular, use or act on the information they received. In ‘2. Having sufficient information to manage my health’, P103 (Strongly Agree) felt he had good information about his health because he could talk with his GP, ask questions and get the answers, and check books and the Internet. His clinician’s perspective (Disagree) was that although he had access to good information, *he doesn’t take it on board, he doesn’t act on it*, which indicated that she felt he only had good information if he used it to improve his health.

Discordance in ‘9. Understand health information well enough to know what to do’ was due to different expectations about patients’ abilities to understand and, from the clinicians’ perspectives, comply with health instructions and information (P115). In ‘3. Actively managing my health’, discordance was about the extent to which setting a goal, or making plans to be healthy, was seen by patients as actively managing their health, yet clinicians wanted to see patients actively carrying out the goal or plan (P105).

### Sub-theme 3b) Patients don’t always know what they don’t know

Discordance in ‘6. Ability to actively engage with healthcare providers’ emerged when clinicians expected patients to manage interactions with healthcare providers differently from the way they often did. The clinicians sometimes attended medical appointments with their patients, and reported that their patients didn’t always know about gaps in their knowledge and so they didn’t know what to ask healthcare providers. In response to an item about asking healthcare providers questions to get information, P110 (Very Easy) said that she asks healthcare providers to explain information in plain language until she understands it and that this sometimes takes time. Her clinician C110 (Quite Difficult) said *she wouldn’t be able to instigate the questioning because she doesn’t know what she doesn’t know*. Although patients tended to say it was easy to discuss things with their doctors, the clinicians said that although patients might have a friendly chat with their doctors, they did not ask questions (P112, P116), did not always understand their health issues and did not leave the consultation with useful information about their health (P103, P113).

### Sub-theme 3c) There are different points of comparison (providers compare across patients, patients compare across providers)

In ‘7. Navigating the healthcare system’, when asked about finding the ‘right’ or ‘best’ care, P114 (Quite Difficult) compared having many healthcare professionals with his preference to have one who got to know him well: *I get a dozen of them [health professionals] in the week and they are all different and not the same one all the time and it is very hard to understand them. If it was the same person coming all the time then you get to know them and understand everything and they would understand the situation.* The clinical perspective of C114 (Quite Easy) was that, compared with other patients, this patient’s complex healthcare needs required a range of healthcare professionals to attend him: *He has the right healthcare because of the severity of his healthcare needs. He doesn’t fall through the gaps. He just has to get to his appointments*.

### Sub-theme 3d) There are different expectations for support when ill

Discordance in ‘4. Social support for health’ revealed different expectations between patients and clinicians for the level of social support and understanding thought reasonable to expect and that could be considered good support (P102, P107, P111). P113 (Strongly Disagree) said *Yeah. It’s hard for family members or anybody to understand that unless they are really in the same situation or have really studied the illness…I find it very, very hard for anybody else to understand the same thing that I’m going through.* C113 (Agree) could see that the family tried to understand his situation: *I think so. I think they try. His family. I’ve only seen him really, really sick a couple of times and they have been very supportive.*

### Theme 4. Different perspectives about a patient’s reliance on healthcare providers

Discordance in this theme was centred on patients and clinicians knowing that patients relied on their healthcare providers to provide and explain information and treatment to them. Patients considered this as knowing where to get health information, being able to appraise health information, and knowing what to do and where to go. The clinicians’ perspective was that patients could not do any of this without the help of a healthcare provider.

In ‘7. Navigating the healthcare system’, patients relied on healthcare providers to tell them what to do and which services to use, and their clinicians knew this (P116). In ‘8. Ability to find good health information’, discordance was due to patients seeking or relying on receiving health information from their known and trusted healthcare providers, with clinicians knowing that they would not search further afield (P104).

Patient narratives in ‘5. Appraisal of health information’ usually explained that they accepted what their healthcare providers told them about health information or that, if they had questions, they asked their trusted healthcare providers. The clinicians’ responses were mostly that the patients could not appraise information by themselves and that they either didn’t do it or needed help to do it. P111 (Strongly Agree) said *I just believe in my GP and specialist. I’m not sure if the information is correct or not. I don’t have a way to look up if the information is correct or not*. C111 (Disagree) said that this patient wouldn’t know how to check if information was right for her or not.

## Discussion

It is incumbent on researchers to demonstrate that the measurement tools they create and use are accurate and fit for their intended purpose [1, 2]. In this study, we worked with people who were disadvantaged and living with complex medical and social situations, many with low education. When asked what they mean by the way they scored the HLQ questions, patients’ narratives matched the intent of items in the majority of cases. These data have important implications for health workers applying the questionnaire in settings where respondents have low socioeconomic position and/or high comorbidity of disease and possibly low health literacy. Alongside robust psychometric studies [14], these qualitative data provide further evidence that the HLQ items and constructs are understood as intended. With this face and construct validity confirmed, researchers, policymakers and funders can have confidence in decisions about projects and programs generated from HLQ data collected at the group and population levels.

This study has also generated new information about the HLQ at the individual patient level by comparing how patients view their health literacy with how clinicians view their patients’ health literacy. A key finding was that clinicians read the words of HLQ items more literally than patients (perhaps because of their technical training and because they have the breadth of experience of the situations of many patients). In addition, patients and their clinicians sometimes have different perspectives about patients’ evolving circumstances; have different expectations for and apply different criteria to assigning scores to some aspects of a patient’s health literacy; and, in terms of health literacy, have different interpretations of patients’ reliance on healthcare providers. These findings have important implications for the use of data derived from a PROM that is used to make assertions about the health literacy status of individual patients.

The data from this study revealed that a clinician can have a perspective about a patient’s health literacy status that differs from the patient’s perspective. This is of clinical importance because, in a small number of instances, if a clinician took the patient’s HLQ score at face value (that is, interpreting it through their own view of the patient’s health context) then opportunities for social and clinical support could be lost. If a patient’s HLQ scores differ from those that a clinician might expect then this can facilitate discussions with the patient. As one set of rich information about a patient’s health literacy status, HLQ data should be triangulated with other data such as patient history, direct observation and clinician intuition.

Some HLQ scales appear to show strong similarities between patient and clinician perspectives (concordance). The clinicians engaged in this study were specifically selected because, as case managers, they were deeply connected with their patients (e.g., consultations in the home, attending clinical appointments with the patients) and they had a good understanding of their patients’ health and health contexts. In other clinical and social settings, clinicians do not have the opportunity to acquire this depth of knowledge – at least not over relatively short periods (i.e., months) – and so their perspectives may, in fact, be even less similar. The findings indicate that the HLQ has the potential to be a powerful adjunct to clinical practice. The provision of patients’ HLQ scores to clinicians early in the patient-clinician relationship may hasten the clinician’s knowledge and understanding of patients’ struggles and capacities, particularly when used to facilitate clinical discussions to uncover barriers to patient self-care and to enable a deeper patient engagement with healthcare services.

Discordance between patient and clinician views were most often observed in scales ‘6. Ability to actively engage with healthcare providers’, ‘4. Social support for health’, ‘2. Having sufficient information to manage my health’, ‘9. Understand health information well enough to know what to do’, and ‘8. Ability to find good health information’. At times, patients rated themselves as being able to easily talk with healthcare providers, having the social support they needed, having sufficient information and understanding of information to manage their health, and knowing how to find the information they needed. However, their relative community assets or functional capacity in these areas were often described as weak by clinicians, and that some patients had little social support or ability to engage with health information or health providers. Some patients admitted that they unquestioningly accepted or relied on information from their clinicians (and so they felt had the information they needed), but clinicians reported that the patients had little ability to independently understand information. Even if a patient’s HLQ scores indicate that they have sufficient information about their health, it is important that clinicians do not assume that the patient has a good understanding of that information. Conversely, if a patient’s scale score indicates they do not have sufficient information, it may be that they do not understand the information they have. Reliance on the patient’s perspective in a self-report questionnaire could exclude important opportunities to instigate health-literacy-related interventions early in a patient’s care.

A key component of this study was that the clinicians knew the patients well. This factor allowed for detailed information to emerge about the everyday things that patients do for their health, and also, importantly, the things they do not do for or do not know about their health. The data asserted that a few patients felt that their intentions to do something active for their health was as indicative of them managing their health as of them actually doing it. Consequently, patient HLQs with scores that indicate the patients actively manage their health may be reflecting something other than what the clinician may expect (i.e., a difference between patient and clinician expectations for scale ‘3. Actively managing my health’). Discordance usually indicated situations where the clinician was expressing their need for perceptible outcomes (e.g., behaviour change after the patient has been given information), or they wanted to see concrete goal setting to help patients achieve that behaviour change. This was a difference between patient responses that reflected patients’ expressed intentions and clinician responses that were looking for (but not seeing) action from the patient. It is important to note that this paper does not report on the second set of scores from clinicians (how they think their patients would respond to the HLQ items), which, in some cases, may be the same as the patient’s score, even if the score from their clinical perspective differs. However, these other data answer a different research question about the difference between clinicians’ perspectives of their patients and what they think their patients’ perspectives would be. This is likely to be a valuable future research direction.

The primary technical reason for discordance in this study was when clinicians applied a literal reading to three words within items: sure, all and plenty. These words were designed to contribute to item difficulty within a scale. Part of the challenge of writing psychometric questionnaire items is to generate items that are easy to endorse (i.e., even people with low levels of the trait can easily respond Strongly Agree or Very Easy) through to items that are harder to endorse (i.e., it is difficult to respond Strongly Agree or Very Easy even with a high level of the trait). Each item within a scale earns its place by measuring a different and defined aspect of the scale. The HLQ wording was derived using a grounded approach, which means the items were derived from a wide range of responses to open questions about engagement in health and health services. This conversational style was deliberately used in item construction where community members rarely read these words as absolute.

Given their in-depth knowledge of a patient, and informed by their knowledge of potentially thousands of other patients, a clinician can, at times, assign a level of health literacy to a patient that differs from the patient’s own assessment. The presence of discordance in HLQ scores does not necessarily mean that the patient’s perspective is wrong, nor that the clinician’s perspective is wrong. Rather, their answers may come from different reference points and they may be using different appraisal criteria [27]. To advance the field, provision of training to better detect these differences may assist clinicians to provide improved care.

### Limitations and strengths of this study

The length of time between respondents completing the HLQs and being interviewed was mostly between 3 and 8 weeks. However, two patients were interviewed 12 weeks (P114) and 21 weeks (P104) after completing their HLQs. Reasons for these delays with patients were because interviews were sometimes difficult to schedule because some patients were difficult to contact, which is consistent with our intent to engage participants who would usually be overlooked for research because they are difficult to access. The clinicians explained that some patients had trust issues and would not answer their phones if they did not recognise the incoming number. In some cases, the clinician facilitated contact between a patient and a researcher. P104 was interviewed face-to-face at a Barwon Health site because the patient’s attention span for a telephone interview was limited, and also the patient’s trusted clinician introduced the patient to the researcher (SG) lessening the patient’s concern about not knowing the researcher. Despite the sometimes long period of time between HLQ completion and interview, the narratives of these patients indicated that recall of the scores and why they chose the scores seemed strong. In fact, as an incidental finding, some respondents were able to describe change between the scores they chose when they completed the HLQ and what they would score at the time of the interview, which indicates that the HLQ may be able detect change in health literacy over time.

This study did not obtain data about non-responders, which may be seen as a limitation. However, a response rate of 18 of the 45 patients (40%) who were asked to complete an HLQ is exceptional from this group of people who required extensive assistance from a case manager to cope with their chronic and complex health conditions. This study conducted research never previously undertaken about the use of the HLQ at the individual patient level to assess this as a possible use of the HLQ. Use of the HLQ in other clinical contexts with individual patients will require validation of score interpretation for that context [2]. The outcomes of this study contribute to the growing body of international validation evidence about the use of the HLQ in different contexts.

Another limitation of this study is that the interview schedule grouped HLQ items within their scales, which is not the order in which respondents completed them on the HLQ. This may have caused participants to respond in interview in a way that was different from how they may have responded if the interview questions had followed the order in which the items appear on the HLQ. However, each participant was asked questions from only a selection of scales, so many of the HLQ items would have been omitted from the interview schedule anyway, which would have led to items appearing in a random way, matching neither the HLQ sequencing of items nor the items within the scales. To maintain consistent organisation of items and ensure all items were covered, it was deemed best to conduct the interviews using sets of items within the scales.

An important strength of this study is that it accessed a group of people who are often missed by research. That is, people who are rarely invited to participate in research because of how difficult it is to engage them. These are often people with low or very low health literacy. Further strengths of this study include that the study was conducted in a real world clinical setting using a psychometrically robust PROM.

This research lays the groundwork for further work (already being undertaken by the authors about validation of the interpretations of PROM data) because it is an initial exploration into qualitative validation methods that go beyond the cognitive interviews that are used to support validation of the psychometric properties of PROMs for aggregated population data. These studies also put into practice long-held theories of validity that it is the inferences derived from data that are determined to be valid for each new context, not the properties of the tool itself [6–8].

## Conclusion

The HLQ and the field of health literacy have been identified by global organisations such as the United Nations (UN) and the World Health Organization (WHO) as having the potential to make substantive contributions to public health and health equality [28–31]. Health literacy is now seen as an opportunity to understand and intervene in social inequalities in health. However, much of the recent research in the field is at the group and population levels. Our research demonstrates that the HLQ has measurement veracity at the patient and clinician level. It also indicates the important implications for the depth and quality of care that a patient might receive if clinicians can detect when they perceive a patient’s health literacy to be different from the way the patient sees it. A primary recommendation of this paper is the use of the HLQ to highlight areas of discordance between clinician and patient perspectives. Awareness of these differences in perspectives can pave the way for clinicians to engage in conversation with patients to better understand their health context, and plan well-founded treatment and care solutions that reflect a patient’s individual health literacy challenges and strengths. This study, in line with the validity driven approach, is part of the ongoing development of the web of quantitative and qualitative evidence about the clinical and public health utility of the HLQ.

## Abbreviations

HLQ: Health Literacy Questionnaire
WHO: World Health Organization
PROM: Patient-reported outcome measure
UN: United Nations
